# The Application of Digital Design Combined with 3D Printing Technology in Skin Flap Transplantation for Fingertip Defects during the COVID-19 Epidemic

**DOI:** 10.1155/2021/5554500

**Published:** 2021-06-08

**Authors:** Hui Lu, Hanshu Peng, Ze Peng, Dingxi Liu, Qimei Wu, Rong Liu

**Affiliations:** ^1^School of Medicine, Wuhan University of Science and Technology, No. 1, Huangjia Lake University Town, Wuhan 430065, China; ^2^Institute of Medical Innovation and Transformation, Puren Hospital of Wuhan, 1 Benxi Road, Wuhan 430081, China; ^3^Department of Orthopedics, Puren Hospital Affiliated to Wuhan University of Science and Technology, 1 Benxi Road, Wuhan 430081, China; ^4^School of Mechanical Automation, Wuhan University of Science and Technology, 1 Benxi Road, Wuhan 430081, China; ^5^Wuhan Liu Sanwu Traditional Chinese Medicine Bone Injury Hospital, Xinzhou District, Wuhan 430081, China

## Abstract

**Objective:**

We aimed to evaluate the advantages of preoperative digital design of skin flaps to repair fingertip defects during the COVID-19 pandemic. We combined digital design with a 3D-printed model of the affected finger for preoperative communication with fingertip defect patients under observation in a buffer ward.

**Methods:**

From December 2019 to January 2021, we obtained data from 25 cases of 30 fingertip defects in 15 males and 10 females, aged 20-65 years old (mean 35 ± 5 years). All cases were treated by digitally designing preoperative fingertip defect flaps combined with a 3D-printed model. Preoperative 3D Systems Sense scanning was routinely performed, 3-matic 12.0 was used to measure the fingertip defect area ranging from 1.5 cm × 3.5 cm to 2.0 cm × 5.0 cm, and the skin flap was designed. The flap area was 1.6 cm × 3.6 cm to 2.1 cm × 5.1 cm. CURA 15.02.1 was used to set parameters, and the 3D model of the affected finger was printed prior to the operation. Full-thickness skin grafts were taken from donor areas for repair.

**Results:**

No vascular crises occurred in any of the 25 cases, and all flaps survived. The postoperative follow-up occurred over 3-12 months. All patients were evaluated 3 months after operation according to the trial standard of hand function evaluation of the Chinese Hand Surgery Society. The results showed that 20 cases had excellent outcomes (80%), four cases had good outcomes (16%), and one case had a fair outcome (4%). The excellent and good rate was 96%.

**Conclusions:**

During the COVID-19 epidemic, fingertip defects were treated with preoperative digital design of fingertip defect flaps combined with 3D printing. Precision design saves surgery time and improves the success rate of surgery and the survival rates of skin flaps. In addition, 3D model simulations improve preoperative communication efficiency, and the personalized design improves patient satisfaction.

## 1. Introduction

Soft tissue defects of fingertips are one of the most common hand injuries, which can lead to loss of sensation, reduced general hand function, and decreased quality of life [1]. It is essential to repair the soft tissue of fingertips as early as possible, to restore hand function and ensure the quality of life. Clinical practice has shown that early operation to close the wound surface and strengthen functional exercise is beneficial to future recovery of function [2]. However, the appearance of COVID-19 has complicated the traditional treatment of finger defects. In early December 2019, Chen et al. [3] reported the first case of pneumonia caused by the novel coronavirus infection in Wuhan, Hubei Province. On February 11, 2020, the disease caused by the novel coronavirus was officially named COVID-19 (coronavirus disease 2019) [4]. Since December 2019, the COVID-19 outbreak has spread across the country [5] and the world, with human-to-human transmission of the virus [6]. In order to contain the spread of the disease, person-to-person contact should be minimized to avoid transmission.

The treatment of fingertip defects is an emergency operation that should be conducted as soon as possible. During the COVID-19 pandemic, however, hospitalized patients are required to undergo routine pharyngeal swabs, COVID-19 antibody tests, and chest CT scans to rule out COVID-19. While waiting for the results, each patient must enter a buffer ward for observation. Buffer wards are rooms where a patient is observed without contact with other patients and before being treated in a general ward until COVID-19 infection has been ruled out. During the observation period, no substantial treatment for a surgical patient with a fingertip defect can be performed, missing a critical window and decreasing the probability of recovery of finger function. Thus, it is essential to develop techniques to reduce time prior to surgery for fingertip defect patients.

For the past few years, digital design and 3D printing technology have significantly improved the efficiency of doctor-patient communication [7, 8], because they can be used for preoperative planning and intraoperative navigation [9–11]. During the COVID-19 epidemic, skin flaps can be precisely digitally designed for patients under observation, with minimal contact with the patient, saving time typically used for designing flaps in the traditional way during the operation. These flaps can also be customized according to the needs of patients, improving patient satisfaction. The 3D model is printed simultaneously to simulate the operation and allows for thorough preoperative communication with the patient only once or as few times as possible. Such preoperative design avoids incomplete and repeated communication and improves the efficiency of preoperative communication.

In this study, fingertip defects were treated by digitally designing skin flaps preoperatively, combined with a 3D-printed model of the affected finger, for fingertip defect patients under observation in a buffer ward during the COVID-19 pandemic. This procedure saves time during the operation and improves the success rate of the operation and the survival rate of the flap with minimal preoperative contact with the patient, thereby reducing the risk of COVID-19 transmission. At present, there are few reports on the application of digital design combined with 3D printing technology in fingertip defect flap transplantation during the COVID-19 pandemic. In this study, 25 cases of 30 fingertip defect patients were selected, and good postoperative efficacy was obtained.

## 2. Materials and Methods

### 2.1. Case Inclusion and Exclusion Criteria

Case inclusion criteria were as follows: (1) patients with negative COVID-19 nucleic acid tests in the outpatient department were rechecked with a nucleic acid test, antibody test, and chest CT examination after hospitalization. Before the examination results were obtained, the patients were temporarily placed in a buffer ward. (2) Physical examination identified the patients as having a fingertip defect. (3) A fingertip defect flap was digitally designed preoperatively. (4) 3D-printed models of the affected finger were used for preoperative communication with patients with fingertip defects.

Exclusion criteria were as follows: (1) patients with old fingertip defects and (2) patients with other diseases who cannot tolerate surgery.

### 2.2. Materials

There were 25 cases with 30 fingers in the group of 15 males and 10 females aged 20-65 years old (mean 35 ± 5 years). Injured fingers were as follows: index finger in five cases, middle finger in eight cases, ring finger in ten cases, and little finger in two cases. The defect area of fingertips ranged from 1.5 cm × 3.5 cm to 2.0 cm × 5.0 cm. One-stage flap repair was performed after emergency debridement.

### 2.3. Digital Design and 3D Printing Technology

#### 2.3.1. Scans of Injured Fingers

The 3D Systems Sense software (3D Systems Company, America) was used to assess injured fingers. Geometric resolution was adjusted to the highest level with scanning capacity at the minimum. The scanner was aimed at the patient's hand to accurately capture an image of the hand on the affected side. Uninjured parts of the hand were removed by cutting, materialized to form a 3D hand model, and exported in STL file format. The same procedure was followed for the healthy side of the hand.

#### 2.3.2. Flap Design

**Figure 1:**
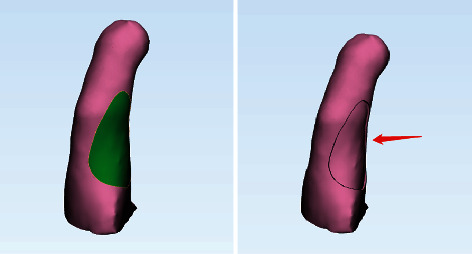
3-matic 12.0 was used to design grafted skin flaps for fingertip defects. The green region simulates the selected donor area; removing the donor flap by pulling out the shell simulates flap cutting.

The STL file was imported into 3-matic 12.0 (Materialise Company, Belgium). First, the healthy hand model was processed, and “Wrap” and “Smooth” were used to smooth the surface of the model. Using the “Mirror” function, a hand model of the affected side was generated. Then, using the same method for the affected hand model, coincident comparison was made between the affected finger of the generated mirrored hand model of the affected side and that of the affected hand model. The extra part is the missing part of the fingertip. The “Trim” tool in “Design” was used to cut the extra part of the affected hand model except the affected finger, keeping the part of the affected finger that needs to be transplanted. The “Mark” tool was used to mark the part of the fingertip defect that needs to be covered, and “Smooth Marking Border” was used for the marked area to generate smooth edges. The generated surface was selected, and the property bar was checked to identify the area of the selected surface, that is, the area of the skin flap of the donor area. According to the needs of the operation, the same area was marked on the side of the affected finger, and the thickness of the shell was adjusted to 2 mm using the “Hollow” tool to extract the flap in the donor area. With the “Boolean Subtraction” tool, the model of the affected finger was subtracted from the model of the skin flap by Boolean subtraction to obtain a model of the fingertip with the defective skin flap (Figure 1), which was exported in STL file format.

#### 2.3.3. Parameters for Designing the 3D Model

The STL file was imported into CURA 15.02.1 (Ultimaker Company, Netherlands), according to a previous study [12], and prior experience was used to set software parameters. The model “Scale” parameter was set to 0.9, while the scale of the printed model was closer to the real size. Because the finger model was wrapped in the design of the skin flap, the model proportions were a little larger than the actual proportions, so when setting parameters, the proportion should be appropriately reduced. The design was then exported in Gcode file format.

#### 2.3.4. 3D Printing

The Gcode-formatted file was imported into a 3D printer (JGAURORA Company, China). Nozzle temperature was set at 210°C, and the hot bed temperature was set at 50°C. After preheating, the 3D-printed model of the fingertip defect of the defective skin flap was obtained.

#### 2.3.5. Preoperative Communication

**Figure 2:**
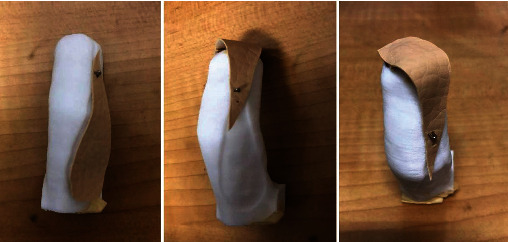
The root of the simulated grafted flap is fixed on the model, and the flipping of the donor flap during the operation is simulated for preoperative communication.

The biological skin flap was pasted on the surface of the 3D model and then cut to obtain the area that fits with the donor area of the defect. This is the transplanted skin flap. The root of the simulated grafted skin flap was fixed on the model, and the rotation was used to simulate the flipping of the donor flap during the operation for preoperative communication (Figure 2).

#### 2.3.6. Intraoperative Guidance

The simulated grafted skin flap was removed from the surface of the 3D model, used to cover the donor area of the patient's finger, and marked on the donor area, to accurately obtain the selected area and range of the donor area intraoperatively before surgery.

### 2.4. Operation Methods

#### 2.4.1. Flap Cut Out

All patients underwent a nucleic acid test, antibody test, and chest CT examination after hospitalization, and the possibility of COVID-19 was excluded. Brachial plexus block anesthesia was performed under a tourniquet, and complete debridement was performed during the operation. The flap was designed on the side of the affected finger, pedicled with the distal end of the digital artery. The distal end of the flap retained 1 cm of soft tissue including the ipsilateral digital vasculature and nerve bundle. The flap area was 1.6 cm × 3.6 cm to 2.1 cm × 5.1 cm. The proximal flap was dissociated in the superficial plane of the vascular nerve bundle from the proximal end to the distal end, the distal 2 cm vascular nerve bundle was dissociated, and the farthest 1 cm vascular nerve bundle was contained in the soft tissue and dissociated with the flap. After the flap was completely dissociated, it was rotated 180° to cover the fingertip defect, and the flap was sutured. Full-thickness skin grafts were taken from donor areas for repair and pressurized bandaging.

#### 2.4.2. Postoperative Management and Follow-Up

Symptomatic supportive treatments such as nerve nutrition, swelling and pain relief, anticoagulation, anti-infection, and antispasticity were routinely conducted. The affected limb was elevated to allow for venous return, and blood circulation was closely observed to prevent and treat vascular crisis. Postoperative wound dressings were actively changed, and drainage strips were removed. Sutures were removed 14 days after the operation, and the plaster cast was removed for external fixation 3 weeks later. The finger joint and wrist joint were strengthened for functional exercise under the guidance of physicians.

A follow-up was conducted within one year after the operation. During the follow-up, the evaluation indexes of the patients' skin flaps were measured and recorded, and the patients were instructed in how to strengthen finger and wrist joints using functional exercises. The standard of hand function evaluation of the Chinese Hand Surgery Society [13] was referenced to comprehensively evaluate the finger flap and function.

## 3. Results

No vascular crises occurred in 25 cases, and all flaps survived. One patient had superficial necrosis of about 0.4 cm × 0.4 cm at the distal end of the skin flap, which gradually healed after the dressing was changed. The postoperative follow-up was 3-12 months. The skin flaps were soft in texture and full in appearance, with good elasticity, no bloat, and color close to normal skin color; partial superficial sensation was restored; and there were no complications such as interphalangeal joint motion disorder or pain in the donor area [14]. The two-point resolution of the skin was 4-8 mm, with an average of 5.1 mm. All patients were evaluated 3 months after the operation according to the trial standard of hand function evaluation of the Chinese Hand Surgery Society. The results showed that outcomes in 20 cases were excellent (80%), four cases were good (16%), and one case was fair (4%). The excellent and good rate was 96%.

### 3.1. Typical Case

**Figure 3:**
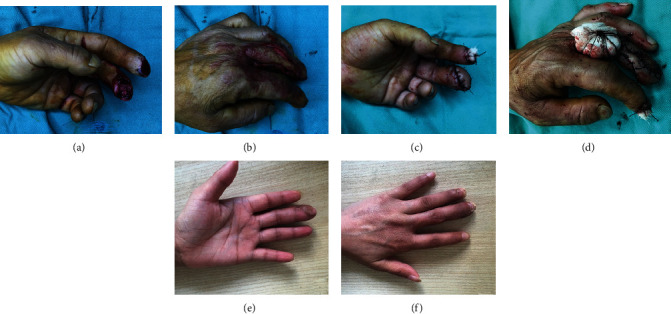
Progression of fingertip replacement prior to surgery and 6 months after surgery: (a) soft tissue defect of the left middle finger before surgery; (b) design of a lateral finger flap; (c) lateral finger flap covering the fingertip defect of the middle finger; (d) full-thickness skin grafts were taken from the donor area for repair and pressurized bandaging; (e, f) good appearance of the skin flap at 6-month follow-up after surgery.

The patient (male, 40 years old) was admitted to the hospital due to soft tissue defect of the left middle finger (Figure 3(a)). Debridement treatment was performed in the first stage, and after 2 days, surgery was performed to repair the wound. The flap was removed according to the preoperative design (Figure 3(b)). The lateral flap of the finger covered the defect of the fingertip of the middle finger, and the blood supply of the flap was good (Figure 3(c)). Full-thickness skin grafts were taken from the donor area for repair and pressurized bandaging (Figure 3(d)). After 6 months of the postoperative follow-up, the skin flap was soft in texture and full in shape, had good elasticity, and was without bloat, and the skin color was close to normal. Some superficial sensation recovered, and no complications such as interphalangeal joint motion disorder and pain at the donor site were found (Figures 3(e) and 3(f)).

## 4. Discussion

### 4.1. Research Status of Fingertip Defect Flap Transplantation

Fingertip skin defects are a common hand trauma, and there are various repair methods, each with advantages and disadvantages [15, 16]. Treatment methods include occluded dressing, local flap transplantation of the same or different fingers, and free flap transplantation [17–22]. Since the 1960s, local advance flap transplantation has been a common method to repair fingertip defects [23]. Fingertip defect flap transplantation technology is constantly optimized and improved based on prior methods, and various new methods emerge, promoting further development. At present, the reverse digital artery island flap, adjacent finger island flap, adjacent finger pedicled flap, dorsal finger fascia flap, or free toe lateral flap are commonly used in clinical repair [24–27], with good curative effect, and traditional surgical techniques have become increasingly mature.

### 4.2. Application of Digital Design Combined with 3D Printing Technology in Skin Flap Transplantation of Fingertip Defects

In recent years, applications of 3D printing technology in the field of medicine have been greatly developed. At present, many domestic universities, medical industry units, and scientific research institutions are constantly increasing investment in the field of 3D printing technology research and have made relatively big breakthroughs. However, there are few reports on the application of 3D printing technology in fingertip defect flap transplantation. Xu et al. [28] collected CT data of hands and feet from seven patients with thumb defects and used digital design combined with 3D printing technology to the select donor area for thumb reconstruction, realizing accurate, personalized, and optimized selection of the donor area of thumb defects. He et al. [29] included 13 cases of proximal interphalangeal joint defects of 13 fingers and simulated the digital test method of repairing facet joint defects with autografts of toe joints and realized accurate reconstruction of facet joints through 3D printing navigation technology, so as to achieve better joint movement function. Zhang et al. [30] selected 60 patients with fingertip defects, 30 with routine abdominal skin flap to repair surgery and 30 with regular 3D design improvement based on the dorsal fascia pedicle retrograde island flap to repair surgery. Through contrast research, Zhang et al. showed that 3D design improved the dorsal fascia pedicle retrograde island flap to repair fingertip defects with better clinical effect, convenient operation, and higher survival rate of the skin flap. In addition, the flap was tolerant to cold, and the flap recovered better after the operation. Mo et al. [31] selected 18 cases of thumb and finger skin defects repaired by free transplantation of toe mini flaps. The 3D images of blood supply in donor areas and defect parameters in recipient areas were analyzed by computer-aided anatomical modeling. Digital 3D reconstruction technology guided surgical design of the donor area, providing a reliable basis for designing a preoperative surgical plan and intraoperative treatment of vascular variation. This procedure reduced the risk during surgery and improved the skin flap survival rate.

At present, preoperative design of fingertip defect flap transplantation is mainly based on the subjective judgment of the surgeon. In traditional procedures, the surgeon covers the affected finger with thin paper or rubber gloves. Based on the experience of the surgeon, the flap area of the donor area is estimated, and the area fitted to the defect area is obtained after cutting the sample cloth. The sample cloth is pasted on the side of the affected finger, and the donor area is marked along the edge of the sample cloth, to design the surgical method and perform the operation. There are some problems in this traditional flap design, such as inaccuracy of donor flap design. The digital surgical design scheme of fingertip defect flap transplantation has thus been adopted, allowing the design to be carried out in a virtual software environment according to the specific injuries of the patients. After printing the 3D physical model of fingertip defects of the defect flap, the biological skin flap is pasted on the surface of the 3D model and then cut to obtain the area that fits with the donor area of the defect. This method can effectively solve the lack of accuracy, scientific basis, and standardization due to subjective experience of previous operations and of the operator in the traditional method. It also effectively avoids a mismatch between the actual cut skin flap and the amputated finger. At the same time, by fixing the root of the simulated grafted skin flap on the model and rotating to simulate turnover of the donor flap during the operation, preoperative communication is more visual and intuitive, which can effectively promote the doctor-patient relationship.

In this study, digital surgical design was adopted to preoperatively design accurate and personalized fingertip defect grafted flaps, according to the specific injuries of different affected fingers. The preoperative surgical design allowed for visualization, which was conducive to smooth operation, reduced unnecessary loss of donor area, and made the selection range of the donor area more accurate, thus improving surgical efficiency. A total of 25 cases were treated with digital design combined with 3D printing defects in the fingertip flap transplantation preoperative design guidance. Intraoperative-specific operation implementation guidance improved accuracy and reduced injury, and after the operation, the repaired finger had cold resistance and the new skin felt good, which supported patient satisfaction.

### 4.3. Advantages and Disadvantages of Digital Design Combined with 3D Printing Technology during the COVID-19 Epidemic

The main advantages of digital design combined with 3D printing during the COVID-19 epidemic are as follows: (1) the use of digital skin flap design for patients in the observation period prior to surgery allows for accurate design of the skin flap in advance with less patient contact, saving time typically required for designing the skin flap in the traditional way during the operation. (2) Designing the flap in advance saves operation time and reduces the time of tourniquet binding on the affected limb, restoring blood supply to the affected finger as soon as possible and improving the survival rate of the flap. (3) Personalized skin flaps can be designed to meet the needs of patients and improve patient satisfaction. (4) Printing a 3D model to simulate surgery is visual and intuitive. It can result in thorough preoperative communication with patients once or as few times as possible, avoiding incomplete and repeated communication and improving efficiency.

The main disadvantages of digital design combined with 3D printing during the COVID-19 epidemic are as follows: (1) it increases the workload of clinicians, and clinicians need to spend time to learn software operations. (2) 3D printing is high cost, making it difficult to popularize in clinical practice.

### 4.4. The Improvement of Digital Design Combined with 3D Printing Technology

At present, the application of digital design combined with 3D printing technology in fingertip defect flap transplantation is relatively rare. It has certain advantages in the aspects of skin flap precision, personalized preoperative design, and preoperative communication. However, for the blood vessels and nerves in the flap transplantation, it is hard to carry out effective preoperative simulation, so more technical means are still needed. Mathes and Neligan [32–34] suggested that CTA (CT angiography) can be used to distinguish the soft tissue plane and clearly show the path of inferior abdominal wall perforating vessels in subcutaneous tissue, subfascial tissue, and intramuscular tissue, which can better guide microsurgery. Combined with CTA technology, microsurgery can be used to locate the route of the donor artery and the starting point of the vessel pedicle, avoid unnecessary damage to the donor area, and improve the survival rate of the donor flap. To solve the problem of poor sensation of skin flap repair, Yang et al. [35] adopted the method of the retrograde island flap with cutaneous branch of the proper finger nerve and digital artery to treat soft tissue defects of the same finger. Feeling in the fingertip recovered well after operation, and the shape was appropriate. In cases where other flaps are not suitable, the reverse island flap containing the cutaneous branch of the proper digital nerve and the digital artery is an alternative method to repair soft tissue defect of the fingertip.

## 5. Conclusion

COVID-19 has complicated the traditional treatment of patients with fingertip defects. Treatment of fingertip defects is an emergency operation that should be conducted as soon as possible. During the COVID-19 pandemic, the time to surgery for patients with fingertip defects is extended. In order to save the treatment time of patients with fingertip defects as much as possible, preoperative digital design of fingertip defect flaps was adopted for patients in the observation period. Preoperative communication was conducted in combination with a 3D-printed model of the affected finger to treat fingertip defects, to achieve the purpose of early treatment with as little patient contact as possible to reduce the risk of transmission. The precise design of the skin flap can save operation time and improve the operation success rate and the survival rate of skin flaps. The efficiency of preoperative communication was improved by printing 3D models to simulate surgery, and the satisfaction of patients was improved by personalized design of skin flaps, providing application value in clinical treatment.

## Data Availability

All data were collected from the Department of Hand and Foot Microsurgery, Puren Hospital, Wuhan City, Hubei Province, China.
